# Polymeric Composites in Road and Bridge Engineering: Characterization, Production and Application

**DOI:** 10.3390/polym15040874

**Published:** 2023-02-10

**Authors:** Wensheng Wang, Yongchun Cheng, Heping Chen, Guojin Tan

**Affiliations:** 1 College of Transportation, Jilin University, Changchun 130022, China; 2 Ingram School of Engineering, Texas State University, San Marcos, TX 78666, USA

As a result of their rapid development, polymer composites are seeing wider use in transportation infrastructure in China and worldwide. Styrene–butadiene–styrene (SBS), epoxy resin (ER), polyethylene (PE), expanded polyethylene (EP), polyurethane (PU), crumb rubber and other polymer materials can be used to modify asphalt and improve the performance of asphalt mix. Meanwhile, the application of polymer admixtures such as PE fiber, polyvinyl alcohol (PVA) fiber, polypropylene (PP) fiber, etc., in bituminous, cementitious or soil materials also improves their strength and durability. Polymer materials are also widely used in pavement maintenance and treatment; they can quickly restore road function and extend the road’s service life.

This Special Issue contains sixteen research papers and one systematic review. The low-temperature properties of SBS-modified asphalt are not satisfactory; thus, Chen et al. analyzed the main factors for improving the low-temperature performance of SBS-modified asphalt base on the orthogonal tests [1]. To solve the insufficient anti-aging performance of modified asphalt with high SBS content, Han et al. added nanomaterials and polyphosphoric acid (PPA) to high SBS content linear-modified asphalt as anti-aging agents [2]. Zhang et al. adopted the mechanochemical method to prepare desulfurized crumb rubber-modified asphalt and analyzed the effects of desulfurization process variables [3]. Furthermore, Xu et al. investigated the modification mechanism, rheological, and aging properties of SBS/desulfurized crumb rubber composite-modified bitumen [4]. Zhao et al. explored the influence of composite modification of activated crumb rubber powder and SBS on asphalt [5]. Gong et al. utilized SBS and rubber crumb to modify the asphalt-based sealants to overcome the poor high-temperature and rheological properties of sealant [6]. SBS in crumb rubber-modified asphalt formed a three-dimensional network structure to improve the performance. Meanwhile, Zhu et al. investigated the fatigue performance of fiber composite modified asphalt mixture [7].

Some research studies are devoted directly to the polymer materials applied in bituminous, cementitious or soil materials. Ren et al. studied the fatigue life and the material design of polymer concrete including ER and PU [8]. Ji et al. analyzed the influence of failure modes of PVA fiber on fiber-reinforced recycled brick powder cementitious composites [9]. Gong et al. investigated the mechanical parameters of basalt fiber polymer-modified active powder concrete (RPC) for the design of structural reinforcement [10]. Due to the high thermal stability, superior mechanical properties, corrosion and chemical resistance of polymer materials, they show great potential for application in road bases, subgrade and embankment slopes, etc. Gong et al. proposed value coefficients to evaluate the cost-effectiveness of PE fiber-reinforced roadbeds and analyzed the effects [11]. Luo et al. used cement, PP fiber and soil curing agent for loess subgrade improvement of a high-speed railway [12]. Wang et al. investigated the improvement effect of PP fiber modified lime-treated soil and fly ash-modified lime-treated soil [13]. Meanwhile, Wang et al. used PP fiber and nano clay to modify lime-treated soil and evaluated the static and dynamic properties of modified lime-treated soil [14]. Jiang et al. used PP fiber and cement to modify iron-ore tailing in road engineering as an effective reuse strategy [15]. Lv et al. adopted waterborne polyurethane (WPU) to reinforce the road demolition waste as a promising new polymer reinforcement material [16].

The Special Issue concludes with a review on fiber-reinforced geopolymers and highlights the difficult aspects of current research, in addition to assessing the literature records [17].

## Data Availability

Not applicable.
